# 

**Published:** 1846-09

**Authors:** 


[We had written the following article and put it aside for the next number, thinking that as we had been under the necessity of presuming on the indulgence of our subscribers for the want of punctuality, it would not be in good taste to ask any farther favors in the present number. Being, however, unexpectedly called upon for more copy at a late hour, we find this fits exactly the vacant niche, and we accordingly place it there. We trust that our subscribers will not think more lightly of the matter for this apology. We assure them it is quite as important to us as if we had not thus yielded to the force of circumstances in reference to it at this time.]

*A few words to subscribers aud friends.*—To talk about one's personal concerns is seldom in good taste, but a little timely explanation may sometimes spare mutual regrets. Will our subscribers and friends indulge us with a few words concerning the Journal and ourselves. In accordance with encouragement received from various sources, and in various ways, we ventured upon our present size and improved appearance. In so doing we assumed, as a matter of course, a considerably increased pecuniary responsibility. Our views and arrangements are such that the Journal will be regularly continued, even should the publication require a pecuniary sacrifice; but the latter, we are free to say, we are very desirous to avoid. It *may* be avoided without a doubt, if they who have felt and expressed a hearty interest in the work, will use their efforts to extend its circulation. Is it asking too much to solicit this at their hands? We are willing to devote a large portion of our time to the labor of editorial duties: we do so cheerfully; and we find in our coadjutors all the alacrity and disinterested zeal that could be desired. In return for these services all that is claimed is, that the success and permanency of the work shall be placed beyond a contingency. This will be at once more than accomplished, if each of our subscribers who feel an interest in its prosperity, will obtain a single additional subscriber. We ask them to think of this, and as the exertions of every one cannot be calculated upon, let all who are disposed, obtain two or more new names, to compensate for the absence of effort by others. How easy for every one to influence by his recommendation at least one acquaintance to subscribe! And how little exertion will this require in comparison with that which we and others have imposed upon ourselves in sustaining the Journal, to say nothing of the pecuniary responsibility. We hope not to be mistaken in these remarks. We have no occasion for complaint or discouragement. Our confidence of success is in no degree impaired. But it is within the power of our friends not only to place the matter at once beyond the possibility of doubt, but to give us the ability to improve still more the character and usefulness of the Journal. We trust that this appeal may lead to results beyond the mere support of the work, and we pledge ourselves to appropriate to its farther enlargement whatever receipts may exceed the absolute cost of publication. Our subscribers and friends, by contributing their exertions to extend its circulation, will thus secure advantages in which they will directly participate. It is believed that there are hundreds of medical men, even in western New York, who either do not subscribe for any medical periodical, or who can easily add another to their list. We have sufficient confidence in the *esprit du corps* of the profession to believe that of these, by far the larger proportion could be easily induced to co-operate in sustaining our enterprise, if the subject were to be personally submitted to their consideration. We can not reach them except through those already our subscribers. Will the latter act as our agents in the business, and render, as we hope to all concerned, a valuable service?

One word more to our patrons, and we have done. We have quite a number on our list who have not yet remitted the amount of subscription for the present volume, and some who are indebted for the first volume. It is proverbially easier to dun for another than on our own account, and it is to be recollected that the sums due are for the exclusive benefit of the printer. Our friends to whose liberality and taste we are indebted for the typographical appearance of the Journal will not be satisfied without realizing something more substantial than empty compliments and prospective reward. If they who are indebted will remit without delay, we promise that it will redound to their advantage, for, between ourselves, we frequently presume on the complaisance of our publishers for the benefit of our readers, and therefore the latter, not less than ourselves, have a direct interest in their continued good nature. We hope the hint just given will be received with a proper spirit, and responded to in such a way that even a hint will again be unnecessary.

---

*Miscellaneous Items.*—The *Western Lancet* has been recently considerably enlarged, and appears bi-monthly, instead of monthly as heretofore. We should have noticed this before, but we failed to receive the two first numbers of the enlarged work. Dr. Lawson, the able editor, is an industrious and vigorous writer, and we infer from the fact that he has been encouraged to increase the size of his work, that his labors as a Journalist are properly appreciated by the profession in his region.

The Illinois Med. and Surg. Journal has, also, been enlarged and improved, and has become a bi-monthly publication. It has also assumed a new title — the *Illinois and Indiana Med. and Surgical Journal.* Dr. Blaney has associated with himself in the editorial department, Drs. Brainard and Herrick of Chicago, and Dr. Evans of Indianapolis — all professors in the Rush Medical College. This Journal will rank with the best conducted and most useful in our country.

We perceive that a new medical periodical is to be commenced in New-York City on the first of October, under the editorship of Dr. Roberts. It is to be entitled the *Annalist,* and to appear bi-weekly. Medical Journalism is getting to be decidedly *the rage;* how is it with readers of medical journals? are they increasing in a corresponding ratio? If so, the increase of Journals in number and size argues well for medical progress.

A paper warfare is going on between Drs. Reyburn and White, of St. Louis, Mo., relative to a suit for *mal praxis* in which the former became involved from the alledged instigation of the latter. We know nothing of the parties, nor have we taken pains to examine into the merits of the controversy from the documents which have been politely forwarded to us, but we are certain of this, that a war of published criminations and recriminations can only result in increased vexation, and in mortification to the individual who is really agrieved, and as certainly tends, in all cases, to bring disrepute upon the profession. Injuries from a professional brother against which a well regulated medical police, and the laws of our country do not afford sufficient protection, had better be borne with silent forbearance, or with quiet contempt. To appeal to the public is only to provoke farther aggression, and excite a contest in which he who is most unscrupulous and impudent will be likely to gather all the laurels. The conscientious combatant, who has truth and justice on his side, is generally, in the end, glad to beat a retreat without obtaining redress, fully satisfied that in such a contest discretion is by far the better part of valor.

Speaking of suits for *mal praxis*, we see reports of fresh cases in almost every number of our exchanges. Whenever we hear of them we infer either that the defendant has importance enough to have bitter enemies, or has been sufficiently successful in acquiring worldly goods to be a mark for cupidity. We recommend, as a preventive, to aspire to but little in the way of professional prominence, and, to be sure not to lay up treasures where "moths and rust will corrupt, and thieves break through and steal."

---

*To Readers and Correspondents.*—We are sorry to be under the necessity of apologising for the tardy appearance of the journal for this month. It has been unavoidably delayed on account of some new arrangements at the establishment at which it is printed. We are assured that it will not be likely to occur again. We appreciate and prize the virtue of punctuality in such matters, and shall do our best to exemplify it in the case of the journal. The publishers have obligingly given an extra number of pages for the present number as an equivalent for its lateness in being issued.

We have received a letter from Dr. Mercer, a young gentleman prosecuting his medical studies in Europe, in which he gives some account of medical men and hospitals at London. It will appear in our next number.

---

*Geneva Medical College.*—To correct an impression which is understood to be somewhat prevalent, that the arrangement of the Geneva school will be affected by the appointment of several of its Professors to chairs in the Buffalo school, we are requested to state that the lectures at Geneva the coming winter will continue as usual (see advertisement). The duties of the Professors in the two institutions will not conflict with each other. In answer to numerous inquiries from various sources relative to the lectures at Buffalo, we would say that a circular will soon be issued and generally circulated.

---

THE BUFFALO MEDICAL JOURNAL
AND MONTHLY REVIEW

Is published monthly at $3,00 per annum, in advance. Subscribers who remit $3,00 will secure the first volume *gratis* with the exception of the sixth number, the copies of which have been mislaid or lost. As the edition of the first volume will soon be exhausted, those who become subscribers without delay will be the gainers in so much as it is desirable to have the work from the commencement.

